# Endothelial Cells Offer a Better Option than Smooth Muscle Cells for the Treatment of Ischemic Limb Disease

**DOI:** 10.3390/cells15171547

**Published:** 2026-08-27

**Authors:** Akazha Green, Hanyu Zhang, Bing Bo, Bijay Guragain, Yalin Wu, Yuhua Wei, Jianyi Zhang, Lei Ye

**Affiliations:** 1 Department of Biomedical Engineering, University of Alabama at Birmingham, Volker Hall, 1670 University Boulevard, Birmingham, AL 35233, USA; angreen@uab.edu (A.G.); zhanghy@uab.edu (H.Z.); bbo@uab.edu (B.B.); bijayg@uab.edu (B.G.); ywu2@uab.edu (Y.W.); yuhua19@uab.edu (Y.W.); 2 Department of Medicine, Division of Cardiovascular Disease, School of Medicine, University of Alabama at Birmingham, Birmingham, AL 35294, USA

**Keywords:** human induced pluripotent stem cells, cell differentiation, smooth muscle cells, endothelial cells, ischemic limb disease

## Abstract

Peripheral arterial disease (PAD) is estimated to affect at least 10 million people in the United States and over 200 million individuals worldwide. The clinical manifestations of PAD range from impaired mobility and pain with activity to chronic limb-threatening ischemia with risk of eventual tissue loss. Despite the significant global prevalence and debilitating consequences of the disease, non-option patients remain largely undertreated. Recognizing that ECs are well-known for their therapeutic role in enhancing perfusion and neovascularization in mouse models of critical limb ischemia, we hypothesize that a synergy of ECs with SMCs may achieve better recovery of perfusion and neovascularization than ECs or SMCs alone. In this study, we compared the therapeutic efficacy of hiPSC-ECs, hiPSC-SMCs, and hiPSC-ECs + hiPSC-SMCs for the treatment of critical limb disease using a NOD-SCID mouse model of hind limb ischemia (HLI). A total of 1 × 10^6^ hiPSC-ECs, 1 × 10^6^ hiPSC-SMCs, or 0.5 × 10^6^ hiPSC-ECs + 0.5 × 10^6^ hiPSC-SMCs were intramuscularly injected into the ischemic limb of the mouse on day 3 after HLI induction. Laser Doppler was recorded on day 0 before, days 3 (i.e., before cell administration) and 31 after HLI induction. Compared with only basal medium injection, hiPSC-ECs alone most effectively improved perfusion (63.8% after normalized non-ischemic limb, *p* < 0.001) and neovascularization (total vessel density = 180.9/magnification, *p* < 0.001) followed by co-administration of hiPSC-ECs + hiPSC-SMCs (perfusion = 59%, *p* = 0.008 and total vessel density = 170.5, *p* = 0.002) compared with basal medium injection (perfusion = 39.7% and total vessel density = 107.2), while hiPSC-SMCs administration achieved mild to moderate improvement (perfusion = 48.9% and total vessel density = 159.5, *p* = 0.01). When profiles of secreted growth factors or cytokines were determined using a protein array, 34 growth factors or cytokines involved in angiogenesis were highly enriched (at least >50% increase) in a hiPSC-ECs conditioned medium, including basic fibroblast growth factor and vascular endothelial growth factor, while there were 14 in hiPSC-SMCs conditioned medium. In conclusion, our results demonstrate that hiPSC-ECs alone most effectively improved perfusion and neovascularization, followed by co-administration of hiPSC-ECs + hiPSC-SMCs, which may be due to the abundance of growth factors and cytokines secreted by hiPSC-ECs. Future studies are warranted to evaluate the therapeutic efficacy of hiPSC-EC and hiPSC-SMC co-transplantation in aged animals with metabolic and cardiovascular comorbidities.

## 1. Introduction

Critical limb ischemia is a severe stage of peripheral artery disease [1]. It causes symptoms such as severe pain, muscle cramping, slow-healing wounds, and ulceration, which may ultimately progress to gangrene [2]. It is associated with a high incidence of serious medical complications, including limb amputation, cardiovascular complications, and death. Almost 1 in 3 people have a limb amputation within 6 months of diagnosis [3,4]. The mortality rate is remarkably high, with approximately 20% of patients dying within 6 months of diagnosis and more than half dying within 5 years [3,5].

Despite advances in medications and interventional surgeries which have shown effectiveness, patients ineligible for revascularization remain an underserved population with limited treatment options. Biological therapies, including cell-based therapies (i.e., endothelial progenitor cells (EPCs) [6,7], endothelial cells (ECs) [8,9,10,11], bone marrow mononuclear cells (BM-MNCs) [12,13], mesenchymal stem cells (MSCs) [14,15,16]) and acellular products such as extracellular vesicles (EVs), secreted from endothelial progenitor cells [17,18] or MSCs [19,20,21], have shown promise for the treatment of ischemic limb diseases in mice.

Previously, we developed a dynamic three-dimensional (3D) culture system in combination with a two-dimensional (2D) culture system to differentiate human induced pluripotent stem cells (hiPSCs) into smooth muscle cells (hiPSC-SMCs) and evaluated their therapeutic potential for the treatment of critical limb ischemia [22]. This study demonstrated that a dynamic 3D environment successfully generated large-scale SMCs, which improved perfusion and neovascularization in mouse ischemic limbs at 2 weeks after treatment. Recognizing that ECs are well-known for their therapeutic role in enhancing perfusion and neovascularization in mouse models of critical limb ischemia [8,9,10,11] and that a synergy of ECs with SMCs may achieve better recovery of perfusion and neovascularization than ECs or SMCs alone [23]. In this study, we aimed to compare the therapeutic efficacy of hiPSC-SMCs, hiPSC-derived ECs (hiPSC-ECs), and a combination of hiPSC-ECs and hiPSC-SMCs for the treatment of critical limb ischemia using a mouse model of hindlimb ischemia. In addition, we examined whether therapeutic efficacy could be maintained for up to 4 weeks after treatment. Surprisingly, our results demonstrated that hiPSC-ECs alone most effectively improved perfusion and neovascularization, followed by co-administration of hiPSC-ECs + hiPSC-SMCs.

## 2. Materials and Methods

### 2.1. Culture and Differentiation of hiPSCs into ECs

The hiPSC-C5 line, which was reprogrammed from cardiac fibroblasts and described previously [22], was used to generate all cell types in this study. Differentiation of hiPSCs into ECs was described previously [9,24] with some modifications. Briefly, hiPSCs at passages 40–45 were cultured in mTeSR Plus media (StemCell Technologies, Vancouver, BC, Canada) for 2–3 days until 20–30% confluence. On day 0, differentiation was initiated by replacing the mTeSR Plus medium with DMEM/F12 medium supplemented with a 100× dilution of B27 Supplement minus insulin (B27-, A1895601, Thermo Fisher, Waltham, MA, USA) and 10 µM CHIR99021 (CHIR; MedChemExpress, Monmouth Junction, NJ, USA) for 24 h. On day 1, the differentiating medium was changed to fresh DMEM/F12 containing a 100× dilution of B27-, VEGF (100 ng/mL), transforming growth factor-beta (TGFβ-1; 10 ng/mL), and erythropoietin (25 ng/mL) for 48 h. On day 3, cells were dissociated into single cells with 0.05% trypsin, harvested, resuspend in endothelial growth medium (EGM) which was composed of EGM2-MV (Lonza, Basel, Switzerland) medium containing 100× dilution of B27, VEGF (100 ng/mL), and SB-431542 (SB; 10 μM, MedChemExpress, Princeton, NJ, USA), and cultured in T-150 flasks that were precoated with fibronectin (1 µg/cm^2^). On day 4, medium would be replaced with fresh EGM supplemented with a 100× dilution of B27, VEGF (200 ng/mL), and SB (15 µM) for 3 days. On day 7, hiPSC-ECs were harvested for analysis or experiments.

### 2.2. Culture and Differentiation of hiPSCs into SMCs

The protocol for the differentiation of hiPSCs into SMCs was described previously [22]. Briefly, hiPSC-C5 was dissociated into single cells, harvested, and cultured in shaker flasks (FPC0125S, TriForest, Irvine, CA, USA) containing 40 mL TeSR™-E8™3D medium (03990, StemCell Technologies, Vancouver, BC, Canada) containing 10 µM Y27632 (HY-10583, MedChemExpress, Princeton, NJ, USA) at 37 °C. Differentiation was initiated on day 0 by replacing the 3D medium with 40 mL RPMI1640 (SH30027.02, Cytiva, Marlborough, MA, USA) medium containing 6 µM CHIR and a 100× dilution of B27-. On day 1, the medium was replaced with 40 mL RPMI1640 medium containing platelet-derived growth factor (PDGF-BB; 10 ng/mL, 10572-H07Y-50, Sino Biological, Wayne, PA, USA), TGFβ (5 ng/mL), and a 100× dilution of B27-. The medium was refreshed on day 3. On Day 5, differentiating cells were harvested, dissociated into single cells, and cultured as monolayers in T-175 flasks containing RPMI1640 supplemented with 4% fetal bovine serum (FBS, SH30910.03, Cytiva, USA), PDGF-BB (10 ng/mL), and TGFβ (5 ng/mL) for 3 days. The medium was refreshed on day 8, and the differentiated SMCs were harvested for subsequent experiments on day 11. This protocol generated hiPSC-SMCs in which >98% of cells expressed smooth muscle actin, >81% expressed SM22, and >89% expressed calponin-1, as assessed by flow cytometry [22].

### 2.3. Flow Cytometry

To determine the differentiation efficiency of ECs, flow cytometry was performed as described previously [9]. Briefly, the differentiated hiPSC-ECs positive for CD31, which was conjugated with fluorescein isothiocyanate (FITC), were assessed using fluorescence-activated cell sorting (FACS). Only stained cells exhibiting the appropriate size and granularity were selected for statistical analysis to determine endothelial differentiation efficiency and for subsequent experiments [9].

### 2.4. Characterization of hiPSC-ECs In Vitro

#### 2.4.1. Fluorescence Immunostaining

Fluorescence immunostaining was used to characterize hiPSC-ECs as described previously [9]. Briefly, ECs were dissociated into single cells, seeded into chamber slides, fixed using 4% paraformaldehyde (PFA), and blocked using UltraV block buffer (TA-125-UB, Thermo Fisher, USA) at room temperature for 7 min. Then, the cells were incubated with 10% donkey serum containing mouse anti-human CD31 conjugated with fluorescein isothiocyanate (FITC) or anti-human CD144 conjugated with phycoerythrin (PE) overnight at 4 °C. The following day, cells were washed in PBS, stained with 4′,6-diamidino-2-phenylindole (DAPI), mounted in Vectashield mounting medium, and examined using an Olympus FV3000 confocal microscope (Olympus, Tokyo, Japan).

#### 2.4.2. Dil-Conjugated Acetylated Low-Density Lipoprotein (Dil-ace-LDL) Uptake

The uptake of Dil-ace-LDL by ECs was assessed as described previously [9]. Briefly, hiPSC-ECs were cultured in EGM supplemented with a 1000× dilution of DAPI overnight and subsequently cultured in EGM supplemented with 10 μg/mL of Dil-ace-LDL (Life Technologies, Carlsbad, CA, USA) at 37 °C for 12 h. Fluorescence images were acquired using an Olympus IX83 fluorescence microscope (Olympus, Tokyo, Japan).

#### 2.4.3. Tube Formation Assessment

Assessment of tube formation by ECs was described previously [9]. Briefly, hiPSC-ECs were seeded into wells of 48-well plates that had been precoated with 150 µL Geltrex (A14133-02, Gibco, Grand Island, NY, USA) and incubated at 37 °C for 24 h. Phase-contrast images of tubular structure were captured using an Olympus IX83 fluorescence microscope.

### 2.5. Assessment of Paracrine Factors Released from Cultured hiPSC-ECs and hiPSC-SMCs

hiPSC-ECs (5 × 10^5^) and hiPSC-SMCs (5 × 10^5^) were cultured in 6-well plates. After washing 3 times with DPBS, the cells were cultured either in 1 mL EBM2 basal medium or 1 mL RPMI basal medium for 48 h. Conditioned medium was collected and submitted to RayBiotech (Norcross, GA, USA) for analysis using the Human Angiogenesis Antibody Array (AAH-CYT-1000).

### 2.6. Murine Hind Limb Ischemia (HLI) Model and Treatment

All animal study protocols were approved by the Institutional Animal Care and Use Committee (IACUC) of the University of Alabama at Birmingham, Birmingham, Alabama, USA. All animal surgical procedures were conducted in accordance with the National Institutes of Health Guide for the Care and Use of Laboratory Animals (NIH publication No. 85–23). In-house-bred, six-month-old male and female NOD-SCID mice were used in all animal experiments. Following surgery, mice were housed at a density of no more than two animals per cage. Animals were maintained in a temperature-controlled environment (25 °C) under a 12 h light/dark cycle with unrestricted access to food and water. To evaluate the relative efficacy of hiPSC-SMCs, hiPSC-ECs, and combined hiPSC-EC/hiPSC-SMC therapy in promoting perfusion recovery in ischemic limbs, HLI was induced in 24 NOD-SCID mice as described previously [9,24] and randomly assigned to 4 animal groups.

Briefly, mice were sedated and anesthetized using 2–3% isoflurane. Hair was removed from the abdomen and rear limbs, and the right limb was disinfected using betadine followed by 70% alcohol. A skin incision was made over the medial aspect of the right hind limb to expose the femoral artery, which was carefully isolated from the surrounding connective tissue along its course from the inguinal ligament to a point just proximal to the patella. The femoral artery and all its branches extending from the inguinal ligament to the bifurcation into the popliteal and saphenous arteries were ligated with 6-0 Prolene sutures (Ethicon, New Brunswick, NJ, USA) to induce hindlimb ischemia. The surgical incision was closed using 6-0 Vicryl sutures (Ethicon, USA), and the animals were allowed to recover under standard housing conditions. Carprofen and buprenorphine were administered for at least 3 days after the surgical procedure. Animals were randomly assigned to four experimental groups (*n* = 6 for each group) on day 3 after surgeries: treatment with 0.1 mL of RPMI1640 medium (i.e., the medium group), or 1 × 10^6^ hiPSC-ECs in 0.1 mL RPMI1640 (i.e., the hiPSC-EC group), 1 × 10^6^ hiPSC-SMCs in 0.1 mL RPMI1640 (i.e., the hiPSC-SMC group), and 0.5 × 10^6^ hiPSC-SMCs + 0.5 × 10^6^ hiPSC-ECs in 0.1 mL RPMI1640 (i.e., the hiPSC-EC + hiPSC-SMC group)_,_ Treatments were administered by four intramuscular injections into the center of the ligated region and the surrounding tissue. Mice exhibiting limb loss or a >50% reduction in limb volume following femoral artery ligation were euthanized according to the predefined humane endpoints. Mice were euthanized by deep anesthesia with 2–3% isoflurane followed by decapitation.

### 2.7. Laser Doppler Imaging

To assess perfusion recovery following HLI induction and treatment, blood perfusion was measured using a laser speckle contrast imaging (LSCI) system (RWD, USA) on day 0 (before surgery as baseline), day 3, and day 31 (i.e., 28 days after treatment) after HLI induction [24]. Briefly, mice were anesthetized with 2–3% isoflurane and placed in a supine position; images of both hind limbs were collected using a Laser Speckle Contrast Imaging System and analyzed. Data from the right ischemic limb were normalized to the corresponding measurements from the left nonischemic limb and presented as percentages.

### 2.8. Immunohistochemistry

Immunohistochemistry analysis of hindlimb tissue was performed as described previously [24,25]. Skeletal muscles from the right ischemic and left nonischemic hind limbs were harvested, embedded in Tissue-Plus O.C.T. Compound (Fisher Scientific, Hampton, NH, USA), and cryosectioned. Cryosections were fixed in 4% PFA, permeabilized with 0.1% Triton X-100, blocked with Ultra V Block, and incubated overnight at 4 °C with rat anti-mouse CD31 antibody (1:100; 553370, BD Biosciences, Franklin Lakes, NJ, USA) and Cy3-conjugated mouse anti-smooth muscle actin (SMA) antibody (1:400; C6198, Sigma-Aldrich, St. Louis, MO, USA) diluted in 10% donkey serum. The following day, sections were washed and incubated with Alexa Fluor 488-conjugated donkey anti-rat IgG (1:500; 712-545-150, Jackson ImmunoResearch, West Grove, PA, USA) for 1 h at room temperature. Sections were then counterstained with DAPI, mounted with VECTASHIELD mounting medium, and coverslipped.

To identify transplanted hiPSC-SMCs and hiPSC-ECs, cryosections were fixed, permeabilized, blocked, and incubated overnight at 4 °C with rabbit anti-human Ku80 antibody (1;100; 2180S, Cell Signaling Technology, Danvers, MA, USA) together with either rat anti-mouse CD31 or Cy3-conjugated anti-SMA antibody. After washing, sections were incubated for 1 h at room temperature with FITC-conjugated donkey anti-rabbit IgG and PE-conjugated donkey anti-rat IgG. Sections were then counterstained with DAPI, mounted with VECTASHIELD, and coverslipped.

To determine arterial vessel maturity, cryosections were fixed, permeabilized, blocked, and incubated overnight at 4 °C with rat anti-CD31, mouse anti-SMA conjugated with Cy3, and rabbit anti-collagen IV. The following day, sections were washed and incubated with Alexa Fluor 488-conjugated donkey anti-rat IgG and Alexa Fluor Cy5-conjugated donkey anti-rabbit IgG (Jackson ImmunoResearch) for 1 h at room temperature. Sections were then counterstained with DAPI, mounted with VECTASHIELD mounting medium, and coverslipped.

Fluorescence images were acquired using an Olympus IX83 fluorescence microscope. Image acquisition and quantitative analyses were performed by investigators blinded to treatment group allocation. CD31-positive capillaries and CD31^+^/SMA^+^ blood vessels were quantified in randomly selected fields at ×20 magnification.

### 2.9. Statistics

Statistical analyses were performed using SPSS version 20.0. Data are presented as mean ± standard deviation (SD). Comparisons within the same group were performed using a paired *t*-test, whereas comparisons between two groups were conducted using an unpaired Student’s *t*-test. Comparisons among three or more groups were analyzed using one-way analysis of variance (ANOVA) followed by Tukey’s post hoc analysis. A *p*-value < 0.05 was considered statistically significant.

## 3. Results

### 3.1. Differentiated hiPSC-ECs Had Typical EC Characteristics

**Figure 1:**
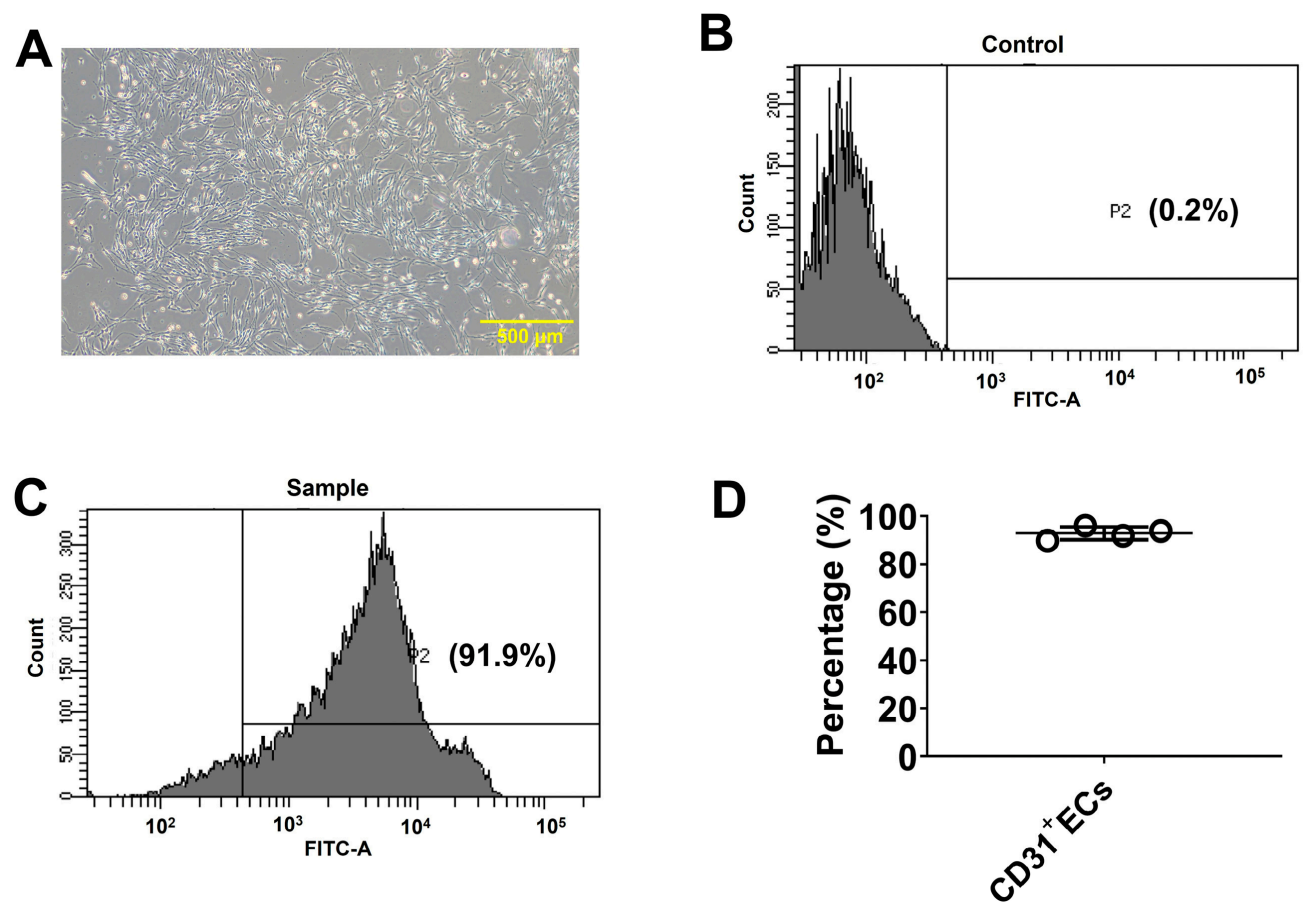
Differentiation efficiency of new modified EC differentiation protocol. (**A**)**.** Morphology of hiPSC-ECs on day 7 after initiating differentiation. Efficiency of hiPSCs into ECs was determined via flow cytometry for CD31 protein expression: (**B**) isotype control and (**C**) CD31 staining. (**D**) The differentiation efficiency based on CD31 protein expression was >90% (Data = mean ± SD, *n* = 4).

**Figure 2:**
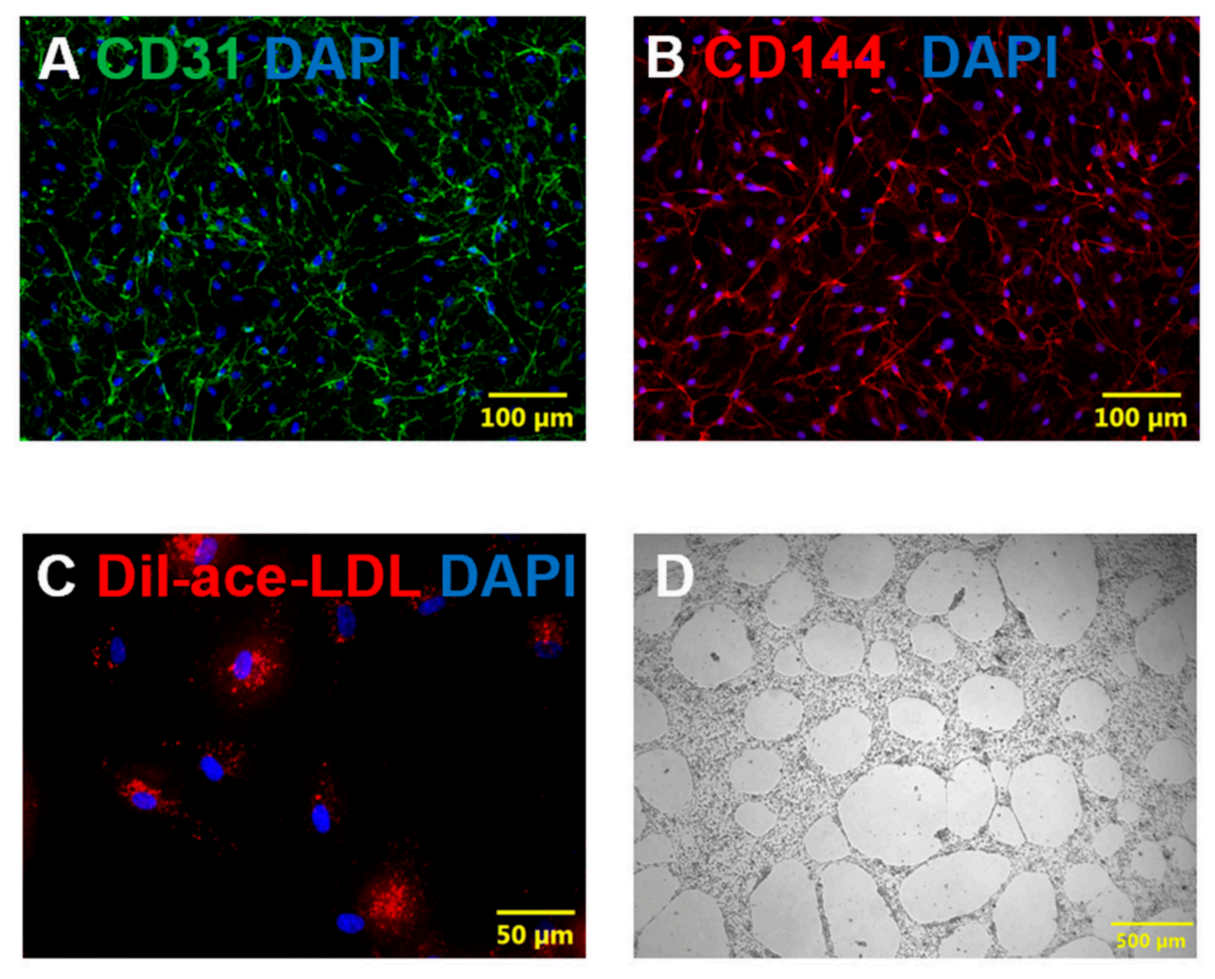
Characterization of hiPSC-ECs. hiPSC-ECs were immunofluorescently stained for CD31 (**A**) and CD144 (**B**) protein expression. To determine the biological function of hiPSC-ECs, Dil-labeled acetylated low-density-lipoprotein (Dil-Ace-LDL) was observed in EC cytoplasm after incubation for 12 h (**C**). (**D**) hiPSC-ECs formed a tubular structure on Geltrex.

The modified hiPSC-EC differentiation protocol was highly efficient (Figure 1). ECs differentiated from hiPSC-C5 had cobblestone morphology (Figure 1A). FACS analyses indicated that the EC-specific marker CD31 was expressed in >90% of differentiated hiPSC-ECs, indicating high purity of ECs differentiated via monolayer differentiation (Figure 1B–D). The purity of hiPSC-ECs was further assessed via immunofluorescence staining, which showed that almost 95% of hiPSC-ECs expressed CD31 and CD144 on the cell membrane (Figure 2A,B).

### 3.2. Differentiated hiPSC-ECs Had Typical Endothelial Cells’ Biological Function In Vitro

In vitro assessments showed that differentiated hiPSC-ECs were able to uptake Dil-ace-LDL and form a tubular structure on Geltrex (Figure 2C,D), indicating that differentiated hiPSC-ECs have biological function in vitro.

### 3.3. hiPSC-EC Transplantation Most Effectively Improved Perfusion in Ischemic Limbs

**Figure 3:**
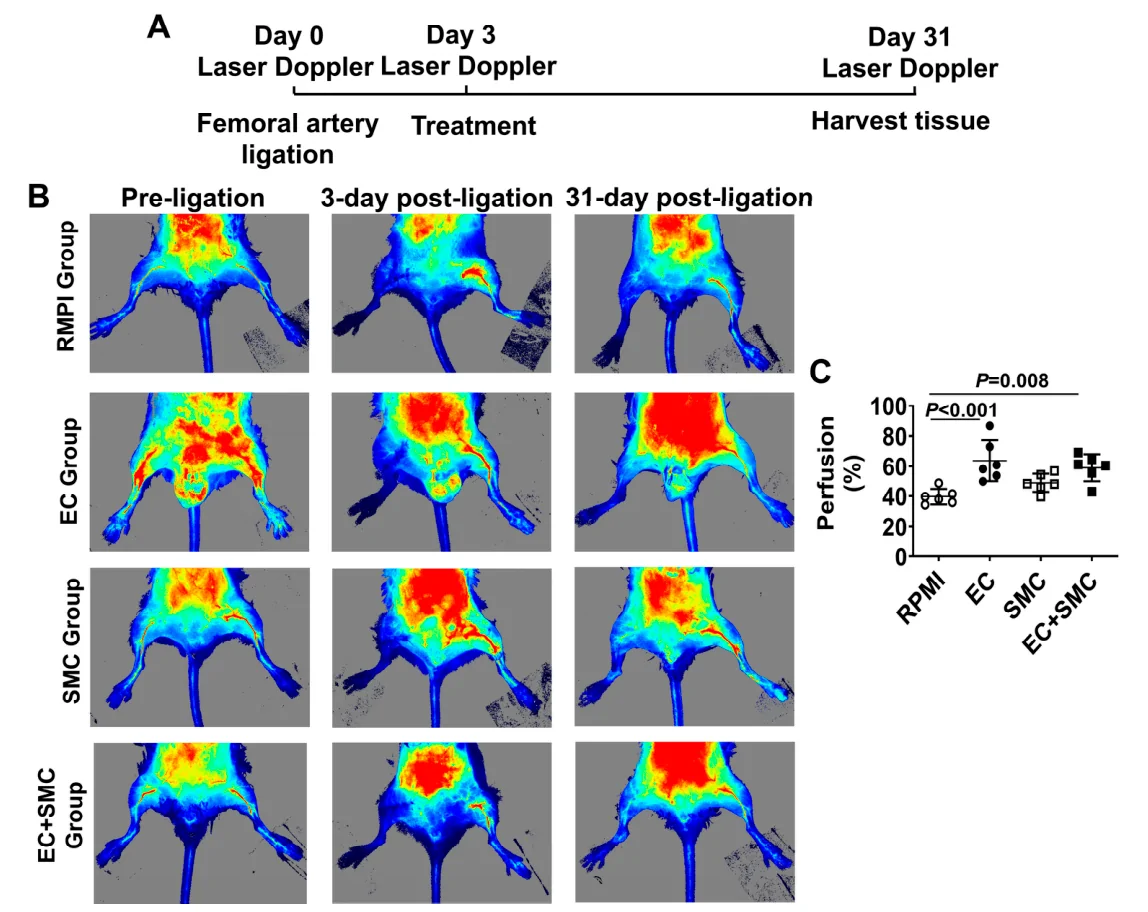
Treatment with hiPSC-ECs most effectively improved perfusion in ischemic mouse limbs. Hindlimb ischemia (HLI) was induced in the right hind limb of NOD-SCID mice, followed by treatment with hiPSC-ECs, hiPSC-SMCs, a combination of hiPSC-ECs and 3D-SMCs, or vehicle control 3 days later. (**A**) A schematic illustrating the timeline of HLI induction, treatment administration, and experimental assessments. (**B**) Representative laser Doppler images were obtained on days 3 and 31 after HLI induction. (**C**) Perfusion on day 31 was quantified as the ratio of blood flow in the ischemic (right) limb to that in the contralateral nonischemic (left) limb and expressed as a percentage. (One-way ANOVA followed by Tukey analysis. Data = mean ± SD. *n* = 6 for each group).

Since the hiPSC-EC differentiation method was efficient, experiments in the mouse HLI model were conducted with the hiPSC-ECs and hiPSC-SMCs. The study included four treatment groups: medium, hiPSC-ECs, hiPSC-SMCs, and hiPSC-ECs + hiPSC-SMCs. No blood perfusion was observed in the ischemic limbs by laser Doppler imaging immediately before treatment administration on day 3 after HLI induction. However, cell transplantation significantly improved perfusion recovery, as evidenced by a higher ischemic-to-nonischemic limb perfusion ratio on day 31 after HLI induction (28 days after treatment) compared with the control group (Figure 3). Significantly improved perfusion was found in mice that received hiPSC-ECs transplantation and hiPSC-ECs + hiPSC-SMCs co-transplantation as compared with mice that received RPMI medium. No significant difference was observed between hiPSC-SMCs and RPMI mouse groups.

**Figure 4:**
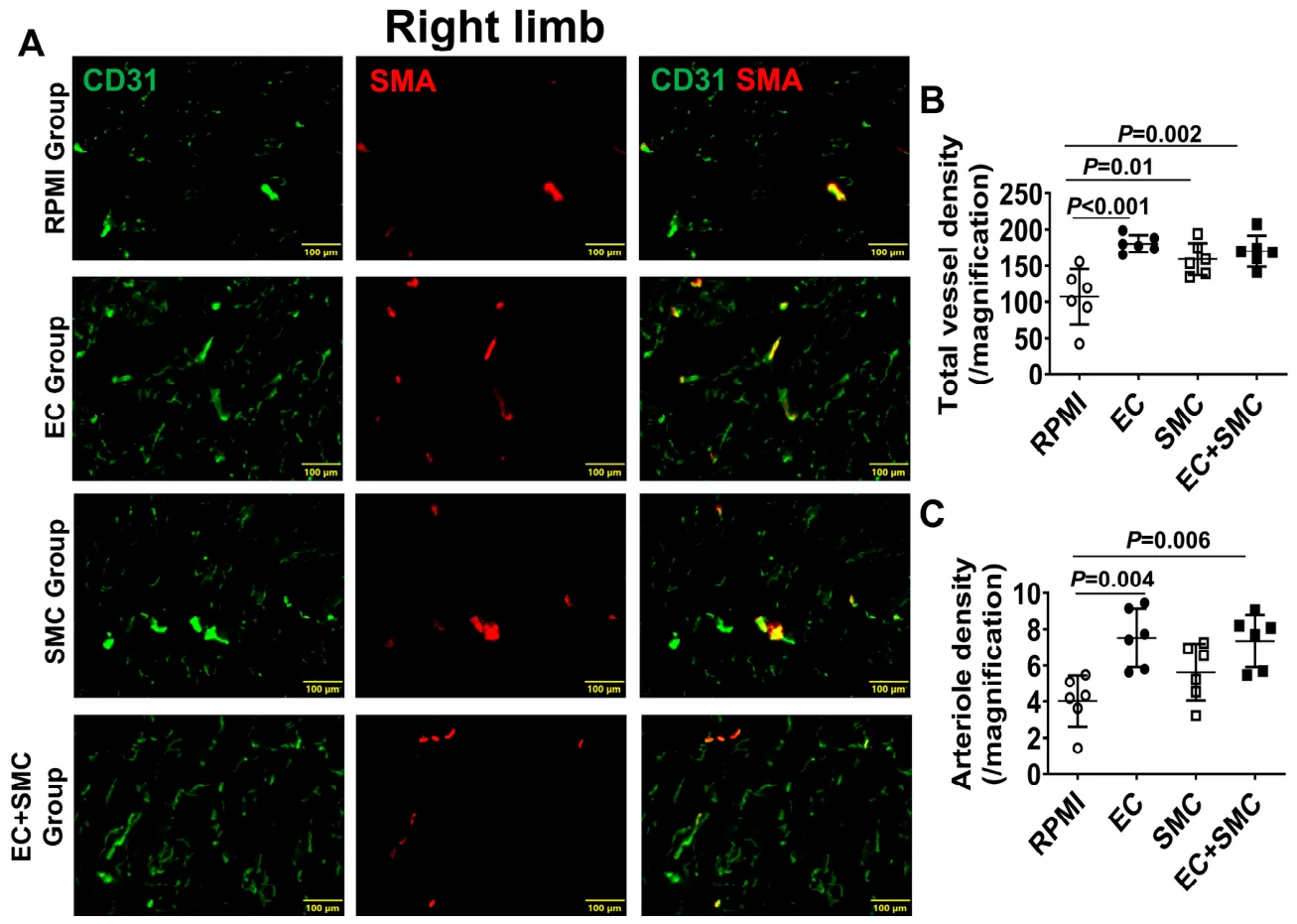

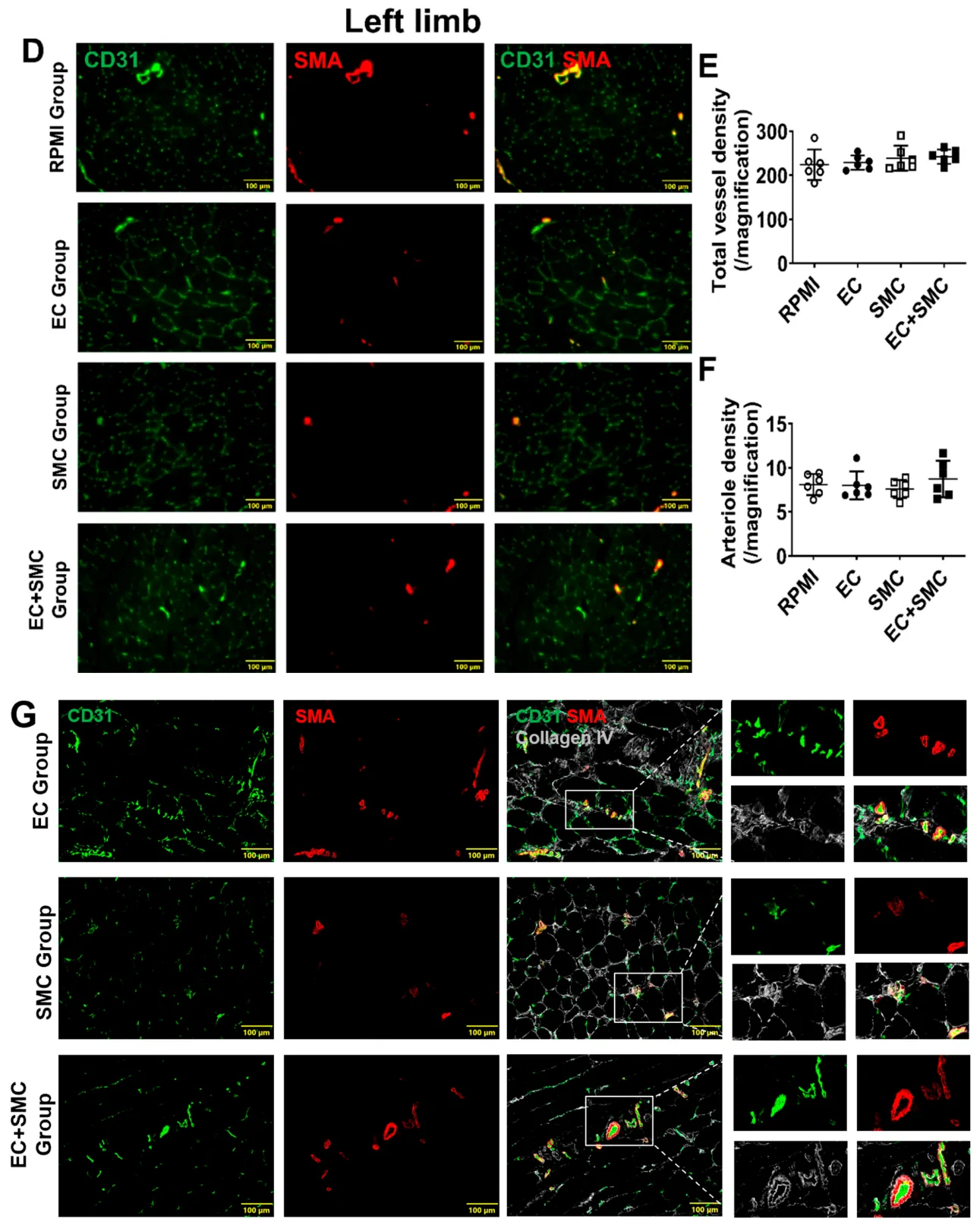
Vascularization quantification in mouse hind limbs. (**A**) Cryosections of the right limbs of mice were immunofluorescently stained for expression of the endothelial marker CD31 conjugated with FITC and smooth muscle marker SMA conjugated with Cy3 at 31 days after femoral artery ligation (i.e., 28 days after treatment). (**B**) Total vessel density was quantified as the number of CD31-positive vessels. (**C**) Arterial density was quantified as the number of vessels positive for both CD31 and α-SMA in the ischemic hind limbs. (**D**) Cryosections of the left limbs of mice were immunofluorescently stained for expression of the endothelial marker CD31 conjugated with FITC and smooth muscle cell marker SMA conjugated with Cy3 at 31 days after femoral artery ligation. (**E**) Total vessel density was quantified as the number of CD31-positive vessels. (**F**) Arterial density was quantified as the number of vessels positive for both CD31 and α-SMA in the ischemic hind limbs. (**G**) Cryosections of the right limbs of mice were immunofluorescently stained for expression of the endothelial marker CD31 conjugated with FITC, smooth muscle marker SMA conjugated with Cy3, and collagen conjugated with Cy5 at 31 days after femoral artery ligation (i.e., 28 days after treatment). The selected areas in white frames in panel G were further magnified. (One-way ANOVA followed by Tukey analysis. Data = mean ± SD. *n* = 6 for each group).

Cryo-sections of skeletal muscle, which were stained for CD31 (detecting all vessels), indicated that all three cell transplantation groups exhibited significantly greater total vessel density in the ischemic limbs than the RPMI control group on day 31 after HLI induction (Figure 4A,B). Furthermore, assessments for arterial density (a vessel expressing both CD31 and SMA) indicated that although all three cell transplantation groups had increased arteriole densities, only hiPSC-ECs and hiPSC ECs + hiPSC-SMCs groups had significantly increased arteriole densities as compared with that in the RPMI group, and no difference was found between hiPSC-SMCs and RPMI groups (Figure 4A,C). Both the total vessel density and arteriole density were similar among all four animal groups in non-ischemic left limbs. Further co-staining for Ku-80 and CD31 or SMA did not detect any cells stained positively for Ku-80 protein expression, indicating that transplanted hiPSC-ECs or hiPSC-SMCs transiently survived in NOD-SCID mouse ischemic muscle and, most probably, that paracrine factors secreted from cells promoted neovascularization.

Co-staining for CD31, SMA, and collagen IV demonstrated basement-membrane collagen IV deposition in nearly all arterial vessels in ischemic muscles at 4 weeks post HLI injury and treatment, indicating a mature vascular phenotype.

Collectively, these observations suggest that hiPSC-EC transplantation most effectively restored perfusion and stimulated neovascularization in mouse ischemic limbs as compared with hiPSC-SMCs or hiPSC-ECs + hiPSC-SMCs transplantation.

### 3.4. hiPSC-ECs Secreted More Abundant Paracrine Factors Involved in Angiogenesis than hiPSC-SMCs Did

To understand why hiPSC-EC treatment achieved better therapeutic outcome, profiles of secreted growth factors or cytokines were determined using protein arrays. Thirty-four growth factors or cytokines involved in angiogenesis showed at least a 50% increase in hiPSC-EC conditioned medium, including basic fibroblast growth factor and vascular endothelial growth factor, whereas only 14 were identified in hiPSC-SMC conditioned medium (Table 1 and Table 2). Further statistical analysis showed that there are six growth factors or cytokines significantly increased in the hiPSC-EC conditioned medium, including epidermal growth factor, insulin-like growth factor-binding protein 2, fibroblast growth factor-9, growth-related oncogene, osteoprotegerin [26], and urokinase-type plasminogen activator receptor, whereas five were identified in the hiPSC-SMC conditioned medium, including angiogenin, brain-derived neurotrophic factors, hepatocyte growth factor, interleukin-8, and macrophage migration inhibitory factor [27].

## 4. Discussion

This study suggests that hiPSC-ECs offer a better option than hiPSC-SMCs or hiPSC-ECs + hiPSC-SMCs for the treatment of critical limb ischemia. Using a modified differentiation protocol, functional hiPSC-ECs can be efficiently differentiated from hiPSCs in 7 days, as evidenced by flow cytometry showing that >90% of hiPSC-ECs expressed CD31, and immunofluorescence staining showed that >95% expressed CD31 and CD144. Differentiated hiPSC-ECs were able to uptake Dil-ace-LDL and form tubular networks on Geltrex. These demonstrate that differentiated hiPSC-ECs have biological function in vitro.

When assessed in a mouse model of HLI, all three cell-based treatments—hiPSC-ECs, hiPSC-SMCs, and the combined hiPSC-ECs + hiPSC-SMCs therapy—improved perfusion recovery to varying degrees. However, only hiPSC-ECs and hiPSC-ECs + hiPSC-SMCs administration achieved significant recovery of perfusion as compared with that in RPMI-injected ischemic limbs.

In addition, our previous study used indocyanine green (ICG) fluorescent angiography, which is a largely qualitative or semi-quantitative measurement, to determine perfusion [22]. The significantly improved perfusion may be due to vessel leakage at 2 weeks after hiPSC-SMCs administration [22]. On the contrary, the current study used Laser Doppler imaging, which measures microvascular blood flow rates quantitatively, to determine perfusion. This may more accurately reflect functional arteriole densities.

Our results challenge a previous study showing that synergistic co-transplantation of hiPSC-ECs and hiPSC-SMCs improved vascular repair and perfusion better than the administration of hiPSC-ECs only [23]. They found that exosomes released from hiPSC-SMCs stimulated migration, proliferation, and tubulation of ECs in vitro. The difference may be due to the following factors. Park et al. generated both hiPSC-ECs and hiPSC-SMCs from purified CD34+ vascular progenitor cells, whereas the hiPSC-ECs and hiPSC-SMCs used in the current study were generated using distinct differentiation protocols. These differences in differentiation strategy may substantially influence EC and SMC phenotypes, including the acquisition of synthetic versus contractile SMC phenotypes, as well as their secretome and EV cargo. In addition, Park et al. found that exosomes secreted by hiPSC-SMCs substantially enhanced the angiogenic activity of hiPSC-ECs. In contrast, our results indicate that hiPSC-ECs exhibit a stronger pro-angiogenic paracrine profile than hiPSC-SMCs. Collectively, these findings suggest that the synergistic effects of EC–SMC co-administration may not be universal but instead depend on the functional phenotypes and paracrine properties of the specific hiPSC-derived EC and SMCs.

Compared with EPCs [6], BM-MNCs [12,13], MSCs [14,28], or their derived EVs, hiPSC-ECs may possess more abundant angiogenic paracrine factors that can directly modulate endogenous EC function and promote angiogenesis, vasculogenesis, and arteriogenesis. However, direct comparative studies are currently limited. Therefore, systematic head-to-head comparisons of hiPSC-ECs, EPCs, and MSCs using complementary in vitro and in vivo approaches are warranted to define their paracrine profiles and determine their relative therapeutic efficacy.

The superior reperfusion and arteriogenesis in the hiPSC-ECs group suggest that hiPSC-ECs alone may be sufficient to repair and regenerate damaged blood vessel networks in ischemic limbs, which mimic critical limb diseases in patients. Although SMCs contribute to vessel stabilization in normal physiological conditions, under ischemic stress, our data suggest that the angiogenic potency of ECs dominates in enhanced perfusion and structural remodeling. This is supported by growth factor and cytokine array results, which showed that thirty-four growth factors or cytokines involved in angiogenesis showed at least a 50% increase in hiPSC-EC conditioned medium, including basic fibroblast growth factor and vascular endothelial growth factor, which are the most potent EC stimulators. These may explain why the highest total vessel density and arteriole density were observed in the hiPSC-ECs administered mouse group, followed by the hiPSC-ECs + hiPSC-SMCs administered mouse group, which were significantly higher than those in the RPMI group. It is worth noting that perfusion recovery in the ischemic limb reached only approximately 60% of that in the non-ischemic limb, suggesting that a single cell administration may be insufficient to completely recover perfusion. Future studies are warranted to determine whether repeated administration of hiPSC-ECs, such as weekly injection, may enhance and sustain perfusion recovery. Alternatively, repeated administration of concentrated hiPSC-EC-conditioned medium may provide a cell-free therapy by leveraging the potent paracrine factors secreted by hiPSC-ECs.

The findings of this study support the therapeutic potential of hiPSC-ECs for no-option patients with PAD. Several limitations of the current study should be acknowledged. First, an immunodeficient mouse model was used to test the efficacy of Xeno transplanted human cells, and therefore it may not fully recapitulate the inflammatory and immune responses that would occur following allogeneic cell transplantation in patients. Future studies using immunocompetent animal models will be necessary to evaluate the potential influence of host immune responses on the therapeutic efficacy. Second, patients with PAD and critical limb ischemia are predominantly older adults and frequently have comorbidities such as diabetes and hypertension [29,30], whereas the present studies were performed in young and healthy mice. Therefore, future investigations in aged animals with metabolic and cardiovascular comorbidities will be important to more rigorously assess the therapeutic efficacy of hiPSC-ECs.

## 5. Conclusions

Compared with hiPSC-SMCs, hiPSC-ECs demonstrated superior therapeutic efficacy by significantly improving perfusion and promoting neovascularization in ischemic muscle. Collectively, these findings underscore the potential of hiPSC-ECs as a promising cell-based therapeutic approach for critical limb ischemia, especially for patients who are ineligible for conventional revascularization procedures.

## Figures and Tables

**Table 1:** Human Cytokine Array C1000-Part 1.

	hiPSC-ECs	hiPSC-SMCs
Angiogenin	2279.87 ± 1547.29	11,883.37 ± 1693.09 ^^,3^
BDNF	11,873.42 ± 5603.37	29,862.73 ± 2805.27 ^^,4^
BLC	11,548.06 ± 3904.97	12,522.08 ± 1054.46
BMP-4	2310.85 ± 2340.73	2432.02 ± 1491.15
BMP-6	134.22 ± 232.47	451.22 ± 781.54 ^
CK beta 8-1	7103.45 ± 3557.59	6835.92 ± 2239.18
CNTF	13,364.37 ± 4237.58	11,750.20 ± 1632.36
EGF	42,246.15 ± 12,083.27 *^,1^	8340.71 ± 2170.12
Eotaxin	4253.50 ± 3242.5 *	788.91 ± 1366.43
Eotaxin-2	1217.30 ± 2108.42	10,907.69 ± 4740.32 ^
Eotaxin-3	5024.51 ± 4161.37	11,594.90 ± 6539.63 ^
FGF-6	10,904.72 ± 3429.68	13,359.56 ± 3923.38
FGF-7	0.00	2249.20 ± 2333.65 ^
Flt-3 Ligand	6916.31 ± 2261.63	11,018.25 ± 3272.7
Fractalkine	12,961.95 ± 4559.73	16,491.33 ± 3035.44
GCP-2	11,652.12 ± 5614.04	17,137.70 ± 892.07
GDNF	26,949.91 ± 7150.64	31,833.21 ± 1746.5
GM-CSF	1135.91 ± 1627.69	4266.61 ± 2473.63 ^
I-309	7924.02 ± 4104.79	11,207.61 ± 3035.64
IFN-gamma	44,346.72 ± 6454.41	41,552.72 ± 4985.43
IGFBP-1	18,949.14 ± 7456.42 *	12,059.78 ± 3103.35
IGFBP-2	123,183.01 ± 9251.04 *^,2^	42,324.67 ± 4713.04
IGFBP-4	14,411.97 ± 8653.68 *	6895.67 ± 3702.43
IGF-I	18,217.20 ± 11,010.82	23,677.96 ± 5152.24
IL-10	2015.45 ± 3490.86	4027.61 ± 4231.05 ^
IL-13	1761.56 ± 2009.74	3467.71 ± 3944.03
IL-15	12,881.64 ± 2717.24	14,081.81 ± 4600.83
IL-16	15,530.44 ± 3203.31	17,733.64 ± 5401.29
IL-1alpha	15,291.49 ± 6614.98	17,602.17 ± 5119.48
IL-1beta	16,356.04 ± 6529.29	21,136.16 ± 2581.33
IL-1ra	31,585.90 ± 8306.77	37,374.63 ± 257.11
IL-2	8472.00 ± 4114.13	13,067.64 ± 2831.7
IL-3	19,644.15 ± 3770.43	21,413.41 ± 3192.18
IL-4	15,700.53 ± 3791.5	17,265.64 ± 6012.73
IL-5	9880.94 ± 3802.59	11,389.91 ± 4031.47
IL-6	12,644.92 ± 4153.08 *	7859.45 ± 3617.71
IL-7	3120.60 ± 3192.09 *	978.72 ± 1695.19
Leptin	5250.75 ± 9021.72	9393.24 ± 4449.31
LIGHT	22,967.72 ± 10,438.15	21,745.13 ± 6177.17
MCP-1	102,488.03 ± 25,123.12 *	63,921.51 ± 3654.61
MCP-2	13,702.53 ± 4161.55	10,887.28 ± 3557.85
MCP-3	8106.89 ± 4349.97	7096.48 ± 3194.72
MCP-4	12,064.87 ± 5772.94	11,808.61 ± 2393.65
M-CSF	23,619.26 ± 7853.48	25,480.57 ± 2647.95
MDC	20,393.66 ± 5736.17	22,653.51 ± 2908.27
MIG	6486.22 ± 3725.51	8704.49 ± 4024.86
MIP-1-delta	10,419.77 ± 3358.56	13,254.76 ± 5729.24
MIP-3-alpha	13,743.09 ± 2469.81	17,440.33 ± 5678.27
NAP-2	21,703.21 ± 2993.78	24,685.61 ± 5998.50
NT-3	23,098.95 ± 3433.91	21,768.30 ± 5896.99
PARC	14,012.57 ± 4344.87 *	7580.16 ± 6940.18
PDGF-BB	2673.23 ± 4630.17 *	1559.11 ± 2700.45
RANTES	12,873.37 ± 11,685.75	9183.51 ± 10,702.45
SCF	15,790.65 ± 7608.05 *	9700.60 ± 9267.76
SDF-1	13,985.36 ± 6327.32 *	8690.35 ± 8446.02
TARC	13,845.00 ± 7340.36	9814.82 ± 7898.9
TGF-beta 1	8648.83 ± 5364.9 *	5752.30 ± 3279.16
TGF-beta 3	12,928.67 ± 6515.44	10,103.69 ± 1720.17
TNF-alpha	7342.97 ± 5180.63	5316.45 ± 1002.02
TNF-beta	6818.74 ± 2951.06 *	3106.23 ± 908.01

* Growth factors or cytokines with levels > 50% higher in the conditioned medium of hiPSC-ECs compared to hiPSC-SMCs; ^ Growth factors or cytokines with levels > 50% higher in the conditioned medium of hiPSC-SMCs compared to hiPSC-ECs; ^1^: *p* = 0.009; ^2^: *p* < 0.001; ^3^: *p* = 0.002; ^4^: *p* = 0.008. Independent T-test. *n* = 3.

**Table 2:** Human Cytokine Array C1000-Part 2.

	hiPSC-ECs	hiPSC-SMCs
Acrp30	1805.92 ± 3127.9	1337.72 ± 2317.01
AgRP	11,331.39 ± 6877.58 *	3469.91 ± 2902.33
Angiopoietin-2	151,198.86 ± 5886.77 *	11,146.96 ± 2981.02
Amphiregulin	8863.75 ± 7980.69 *	1183.80 ± 2050.41
axl	7049.16 ± 5851.43	8697.81 ± 1583.79
bFGF	11,410.43 ± 6525.17 *	4584.07 ± 3814.71
Beta-NGF	11,016.40 ± 3759.35 *	3906.33 ± 2723.87
BTC	10,683.60 ± 4691.74 *	4017.22 ± 2445.82
CCL28	4089.99 ± 4156.66 *	1072.97 ± 1019.51
CTACK	30,553.80 ± 3933.69	34,622.31 ± 7854.28
dtk	14,412.84 ± 2969.22	23,525.23 ± 8409.6
EGF-R	3073.38 ± 1030.66	8107.70 ± 3343.05 ^
ENA-78	10,325.96 ± 3295.73	11,538.22 ± 5030.71
Fas/TNFRSF6	9289.37 ± 3437.01	7872.09 ± 3263.05
FGF-4	11,728.71 ± 2889.32	8912.07 ± 3426.54
FGF-9	19,344.89 ± 5748.88 *^,1^	6291.14 ± 2125.34
G-CSF	9237.25 ± 7347.75 *	0.00
GITR ligand	19,026.26 ± 2708.53	12,735.05 ± 6468.77
GITR	20,028.56 ± 2015.96 *	12,025.39 ± 6896.55
GRO	85,298.11 ± 10,726.51 *^,2^	41,148.72 ± 2807.92
GRO-alpha	38,433.74 ± 1757.26	30,123.64 ± 4567.35
HCC-4	24,237.50 ± 3019.41	20,710.11 ± 7099.5
HGF	14,645.90 ± 3479.63	94,705.76 ± 12,458.94 ^^,5^
ICAM-1	5072.36 ± 1331.18	12,753.85 ± 6739.87 ^
ICAM-3	2954.55 ± 2154.18	7039.51 ± 4078.21 ^
IGF-BP-3	13,052.42 ± 1820.86	14,661.98 ± 1580.48
IGF-BP-6	14,636.70 ± 3347.76	22,789.21 ± 2131.71
IGF-I SR	29,723.67 ± 1835.41	24,970.87 ± 5684.25
IL-1 R4/ST2	6980.80 ± 1105.79	6820.06 ± 2354.04
IL-1 RI	14,034.98 ± 3863.41	11,887.28 ± 4487.55
IL11	23,254.20 ± 3556.46	19,048.18 ± 4046.93
IL12-p40	11,702.62 ± 2088.01	9433.69 ± 5598.2
IL12-p70	13,375.62 ± 1470.05 *	8232.93 ± 5801.87
IL17	8875.17 ± 2337.82 *	5402.89 ± 3620.41
IL-2 Ra	29,534.63 ± 2936.45	23,902.92 ± 9793.4
IL-6 R	21,393.17 ± 2422.42	28,462.95 ± 1946.2
IL8	38,474.63 ± 4861.83	96,928.08 ± 3546.57 ^^,6^
I-TAC	22,439.53 ± 4324.96	29,431.36 ± 10,194.26
Lymphotactin	24,955.16 ± 4348.89	37,132.93 ± 10,456.51
MIF	27,235.30 ± 5230.87	74,348.59 ± 7138.89 ^^,7^
MIP-1-alpha	28,209.64 ± 3300.22	33,940.57 ± 5741.6
MIP-1-beta	22,425.65 ± 2564.08	21,849.89 ± 6397.36
MIP-3-beta	969.08 ± 1678.5	1829.28 ± 1779.23
MSP-a	1575.25 ± 2728.41 *	999.40 ± 1416.9
NT-4	9142.85 ± 4203.47	6352.76 ± 2474.92
Osteoprotegerin	29,571.14 ± 4651.53 *^,3^	19,562.61 ± 1239.26
Oncostatin M	7538.99 ± 2396.92 *	3572.92 ± 1098.59
PlGF	7251.18 ± 1848.36	4879.21 ± 3251.25
sgp130	4338.58 ± 3184.64	6478.68 ± 2233.97
sTNF RII	33,691.42 ± 3856.8	25,915.51 ± 8322.71
sTNF-RI	23,405.66 ± 3632.9	25,958.15 ± 2601.06
TECK	48,971.24 ± 10,562.76	48,009.38 ± 11,050.56
TIMP-1	158,669.95 ± 3375.38	158,623.20 ± 2719.68
TIMP-2	157,553.56 ± 1754.02	158,643.67 ± 2744.94
TPO	23,216.56 ± 5856.75	24,673.92 ± 6715.12
TRAIL-R3	15,229.35 ± 2604.46	11,082.61 ± 8487.93
TRAIL-R4	11,393.77 ± 1617.48 *	5886.84 ± 5274.43
uPAR	31,996.28 ± 1245.83 *^,4^	18,786.58 ± 4870.19
VEGF	6158.70 ± 3413.91 *	1539.79 ± 1400.35
VEGF-D	6505.58 ± 3567.52 *	1412.18 ± 2103.47

* Growth factors or cytokines with levels > 50% higher in the conditioned medium of hiPSC-ECs compared to hiPSC-SMCs; ^ Growth factors or cytokines with levels > 50% higher in the conditioned medium of hiPSC-SMCs compared to hiPSC-ECs; ^1^: *p* = 0.021; ^2^: *p* = 0.002; ^3^: *p* = 0.023; ^4^: *p* = 0.10; ^5^: *p* < 0.001; ^6^: *p* < 0.001; ^7^: *p* < 0.001. Independent T-test. *n* = 3.

## Data Availability

The data supporting the conclusions of this article are included in the manuscript and are available from the corresponding authors upon reasonable request.

## References

[1] Teraa M., Conte M.S., Moll F.L., Verhaar M.C. (2016). Critical Limb Ischemia: Current Trends and Future Directions. J. Am. Heart Assoc..

[2] Kinlay S. (2016). Management of Critical Limb Ischemia. Circ. Cardiovasc. Interv..

[3] Abu Dabrh A.M., Steffen M.W., Undavalli C., Asi N., Wang Z., Elamin M.B., Conte M.S., Murad M.H. (2015). The natural history of untreated severe or critical limb ischemia. J. Vasc. Surg..

[4] Becker F., Robert-Ebadi H., Ricco J.-B., Setacci C., Cao P., de Donato G., Eckstein H., De Rango P., Diehm N., Schmidli J. (2011). Chapter I: Definitions, Epidemiology, Clinical Presentation and Prognosis. Eur. J. Vasc. Endovasc. Surg..

[5] Adam D.J., Beard J.D., Cleveland T., Bell J., Bradbury A.W., Forbes J.F., Fowkes F.G.R., Gillepsie I., Ruckley C.V., Raab G. (2005). Bypass versus angioplasty in severe ischaemia of the leg (BASIL): Multicentre, randomised controlled trial. Lancet.

[6] Sukmawati D., Tanaka R. (2015). Introduction to next generation of endothelial progenitor cell therapy: A promise in vascular medicine. Am. J. Transl. Res..

[7] Lagonda C.A., Lyons C.J., Liu Y., Creane M., O’BRien T. (2026). The role of endothelial colony forming cells in the treatment of limb ischaemia: Challenges in clinical translation. Stem Cells Transl. Med..

[8] MacAskill M.G., Saif J., Condie A., Jansen M.A., MacGillivray T.J., Tavares A.A., Fleisinger L., Spencer H.L., Besnier M., Martin E. (2018). Robust Revascularization in Models of Limb Ischemia Using a Clinically Translatable Human Stem Cell-Derived Endothelial Cell Product. Mol. Ther..

[9] Su L., Kong X., Lim S., Loo S., Tan S., Poh K., Dutton J., Stewart C., Cook S., Su X. (2018). The prostaglandin H2 analog U-46619 improves the differentiation efficiency of human induced pluripotent stem cells into endothelial cells by activating both p38MAPK and ERK1/2 signaling pathways. Stem Cell Res. Ther..

[10] Deng D., Zhang Y., Tang B., Zhang Z. (2024). Sources and applications of endothelial seed cells: A review. Stem Cell Res. Ther..

[11] Rufaihah A.J., Huang N.F., Jamé S., Lee J.C., Nguyen H.N., Byers B., De A., Okogbaa J., Rollins M., Reijo-Pera R. (2011). Endothelial Cells Derived From Human iPSCS Increase Capillary Density and Improve Perfusion in a Mouse Model of Peripheral Arterial Disease. Arter. Thromb. Vasc. Biol..

[12] Saigawa T., Kato K., Ozawa T., Toba K., Makiyama Y., Minagawa S., Hashimoto S., Furukawa T., Nakamura Y., Hanawa H. (2004). Clinical Application of Bone Marrow Implantation in Patients With Arteriosclerosis Obliterans, and the Association Between Efficacy and the Number of Implanted Bone Marrow Cells. Circ. J..

[13] Teraa M., Sprengers R.W., Schutgens R.E., Slaper-Cortenbach I.C., van der Graaf Y., Algra A., van der Tweel I., Doevendans P.A., Mali W.P., Moll F.L. (2015). Effect of Repetitive Intra-Arterial Infusion of Bone Marrow Mononuclear Cells in Patients With No-Option Limb Ischemia. Circulation.

[14] Navarro L.V.L., Chen X., Viviescas L.T.G., Ardila-Roa A.K., Luna-Gonzalez M.L., Sossa C.L., Arango-Rodríguez M.L. (2022). Mesenchymal stem cells for critical limb ischemia: Their function, mechanism, and therapeutic potential. Stem Cell Res. Ther..

[15] Liew A., O’Brien T. (2012). Therapeutic potential for mesenchymal stem cell transplantation in critical limb ischemia. Stem Cell Res. Ther..

[16] Song Y., Zhang T.-J., Li Y., Gao Y. (2020). Mesenchymal Stem Cells Decrease M1/M2 Ratio and Alleviate Inflammation to Improve Limb Ischemia in Mice. Med. Sci. Monit..

[17] Zhao C., Xing Z., Wei X., Liao G., Yang D., Liu H., Fan Y. (2023). Multibiofunctional TFEB-engineered endothelial progenitor cell-derived extracellular vesicles/hydrogel system for rescuing critical limb ischemia. Chem. Eng. J..

[18] Terriaca S., Fiorelli E., Scioli M.G., Fabbri G., Storti G., Cervelli V., Orlandi A. (2021). Endothelial Progenitor Cell-Derived Extracellular Vesicles: Potential Therapeutic Application in Tissue Repair and Regeneration. Int. J. Mol. Sci..

[19] Gangadaran P., Rajendran R.L., Lee H.W., Kalimuthu S., Hong C.M., Jeong S.Y., Lee S.-W., Lee J., Ahn B.-C. (2017). Extracellular vesicles from mesenchymal stem cells activates VEGF receptors and accelerates recovery of hindlimb ischemia. J. Control. Release.

[20] Rezaie J., Nejati V., Mahmoodi M., Ahmadi M. (2022). Mesenchymal stem cells derived extracellular vesicles: A promising nanomedicine for drug delivery system. Biochem. Pharmacol..

[21] Peng X., Liu J., Ren L., Liang B., Wang H., Hou J., Yuan Q. (2023). Extracellular vesicles derived from hypoxia-preconditioned bone marrow mesenchymal stem cells ameliorate lower limb ischemia by delivering miR-34c. Mol. Cell. Biochem..

[22] Green A., Wei Y., Warram J.M., Hartman Y.E., Geng X., Nguyen T., Ye L., Zhang J. (2024). Dynamic three dimensional environment for efficient and large scale generation of smooth muscle cells from hiPSCs. Stem Cell Res. Ther..

[23] Park J.J., Kwon Y.W., Kim J.W., Park G.T., Yoon J.W., Kim Y.S., Kim D.S., Kwon S.M., Bae S.S., Ko K. (2021). Coadministration of endothelial and smooth muscle cells derived from human induced pluripotent stem cells as a therapy for critical limb ischemia. STEM CELLS Transl. Med..

[24] Su L., Kong X., Loo S.J., Gao Y., Kovalik J.-P., Su X., Ma J., Ye L. (2021). Diabetic Endothelial Cells Differentiated From Patient iPSCs Show Dysregulated Glycine Homeostasis and Senescence Associated Phenotypes. Front. Cell Dev. Biol..

[25] Wei Y., Green A., Guragain B., Bo B., Jiang Y., Zhu H., Zhang J., Ye L. (2025). Human induced pluripotent stem cell-derived nanovesicles for the treatment of ischemic limb disease. Acta Biomater..

[26] McGonigle J.S., Giachelli C.M., Scatena M. (2008). Osteoprotegerin and RANKL differentially regulate angiogenesis and endothelial cell function. Angiogenesis.

[27] Asare Y., Schmitt M., Bernhagen J. (2013). The vascular biology of macrophage migration inhibitory factor (MIF). Thromb. Haemost..

[28] Kang W.C., Oh P.C., Lee K., Ahn T., Byun K. (2016). Increasing injection frequency enhances the survival of injected bone marrow derived mesenchymal stem cells in a critical limb ischemia animal model. Korean J. Physiol. Pharmacol..

[29] Houghton J.S.M., Saratzis A.N., Sayers R.D., Haunton V.J. (2024). New Horizons in Peripheral Artery Disease. Age Ageing.

[30] Cutmore C., Aitken S. (2022). Managing Chronic Limb-Threatening Ischaemia in Patients at the Extremes of Older Age Requires a Patient-Focused Approach. World J. Surg..

